# Prognostic significance of MCM2, Ki-67 and gelsolin in non-small cell lung cancer

**DOI:** 10.1186/1471-2407-6-203

**Published:** 2006-08-01

**Authors:** Jun Yang, Nithya Ramnath, Kirsten B Moysich, Harold L Asch, Helen Swede, Sadir J Alrawi, Joel Huberman, Joseph Geradts, John SJ Brooks, Dongfeng Tan

**Affiliations:** 1 Roswell Park Cancer Institute, Buffalo, NY 14263, USA; 2 Connecticut Tumor Registry, Department of Public Health, Hartford, CT 06134, USA; 3 Department of Pathology, Duke University, Durham, NC 27710, USA; 4 Dept. of Pathology and Laboratory Medicine, University of Pennsylvania, Philadelphia, PA 19104, USA; 5 Dept. of Pathology and Lab Medicine, University of Texas Health Science Center, Houston, TX 77030, USA

## Abstract

**Background:**

Uncontrolled proliferation and increased motility are hallmarks of neoplastic cells, therefore markers of proliferation and motility may be valuable in assessing tumor progression and prognosis. MCM2 is a member of the minichromosome maintenance (MCM) protein family. It plays critical roles in the initiation of DNA replication and in replication fork movement, and is intimately related to cell proliferation. Ki-67 is a proliferation antigen that is expressed during all but G_0 _phases of the cell cycle. Gelsolin is an actin-binding protein that regulates the integrity of the actin cytoskeletal structure and facilitates cell motility. In this study, we assessed the prognostic significance of MCM2 and Ki-67, two markers of proliferation, and gelsolin, a marker of motility, in non-small cell lung cancer (NSCLC).

**Methods:**

128 patients with pathologically confirmed, resectable NSCLC (stage I-IIIA) were included. Immunohistochemistry was utilized to measure the expressions of these markers in formalin-fixed, paraffin-embedded tumor tissues. Staining and scoring of MCM2, Ki-67 and gelsolin was independently performed. Analyses were performed to evaluate the prognostic significance of single expression of each marker, as well as the prognostic significance of composite expressions of MCM2 and gelsolin. Cox regression and Kaplan-Meier survival analysis were used for statistical analysis.

**Results:**

Of the three markers, higher levels of gelsolin were significantly associated with an increased risk of death (adjusted RR = 1.89, 95% CI = 1.17–3.05, p = 0.01), and higher levels of MCM2 were associated with a non-significant increased risk of death (adjusted RR = 1.36, 95% CI = 0.84–2.20, p = 0.22). Combined, adjusted analyses revealed a significantly poor prognostic effect for higher expression of MCM2 and gelsolin compared to low expression of both biomarkers (RR = 2.32, 95% CI = 1.21–4.45, p = 0.01). Ki-67 did not display apparent prognostic effect in this study sample.

**Conclusion:**

The results suggest that higher tumor proliferation and motility may be important in the prognosis of NSCLC, and composite application of biomarkers might be of greater value than single marker application in assessing tumor prognosis.

## Background

Abnormal cell proliferation, which results from deregulation of the cell cycle, is fundamental in tumorigenesis. The integrated mechanisms that regulate the accurate replication of DNA and correct division of cells are thus pivotal in the neoplastic process [1,2]. Regulation of the cell cycle is complex and involves a wide variety of genes and proteins, among which the minichromosome maintenance (MCM) nuclear proteins are essential replication initiation factors. The MCM protein family consists of six major isoforms (MCM2-7), which have similar biochemical functions [3] and are equally important for continuous chromosome replication after the activation of early origins of DNA replication [4]. During the cell cycle, the MCM proteins form a hexameric complex, which is a key component of the prereplication complex that assembles at replication origins during early G_1 _phase [3,5]. MCM proteins restrict DNA synthesis to only once per cell cycle [3,4,6], and regulate DNA elongation [4]. These functions of MCM proteins imply that they are correlated with cell proliferation, which has been consistently supported by experimental evidence. For example, the onset of DNA synthesis was inhibited if anti-MCM2 antibody was injected into cells during G_1 _[6], and depletion of MCMs after initiation irreversibly blocked the progression of replication forks [4]. Moreover, MCM2 expression in developing *Drosophila *embryos followed a pattern corresponding to the fast dividing cells, and inactivation of MCM2 inhibited cell proliferation [7]. Cellular expression of MCMs was constitutively high in proliferating cells but low or undetectable in quiescent cells [3,8,9].

Ki-67, another important cell proliferative biomarker, is a nuclear protein containing phosphorylation sites for a variety of kinases, putative nuclear targeting sequences, and a forkhead-associated domain, similar to those of cell-cycle-regulating proteins [10-12]. During mitosis, phosphorylation and dephosphorylation of Ki-67 occur at the breakdown and the reorganization of the nucleus, two hallmark occasions of the cell cycle. These posttranslational modifications are accompanied by the redistribution of Ki-67 from the interior of the nucleus to the periphery of the condensed chromosomes and vice versa [12,13]. Although recognized as a cell cycle regulating protein, the specific functions of Ki-67 remain elusive, mainly due to its lack of homology with other proteins [13]. Some proposed functions of Ki-67 include organization and maintenance of the architecture of DNA, and synthesis of ribosomes during mitosis [14,15]. Since Ki-67 is expressed during all phases of the cell cycle except G_0_, cellular expression of Ki-67 has provided a measure of tumor proliferation [16,17]. The prognostic value of Ki-67 has been reported in various tumors, including cancers of breast, soft tissue, lung, cervix, prostate, and brain [17-21].

Gelsolin is the most potent actin binding and severing protein identified to date which is widely expressed in mammalian tissues [22,23]. It regulates the integrity and dynamics of actin cytoskeleton and maintains the proper cellular morphological structure and motility through severing, capping and nucleating actin filaments [23-25]. Knockout animal models have clearly demonstrated the critical role of gelsolin in facilitating cell motility [26-30]. An inverse association between gelsolin expression and patient survival time has been observed in lung cancer and breast cancer [31-33], suggesting cellular motility mediated through gelsolin expression plays an important role in the progression and prognosis of malignant tumors.

Although MCM2, Ki-67, two markers of cell proliferation, and gelsolin, marker of cell motility, have been previously studied, no study has been conducted assessing the potential combined prognostic effect of these markers in non-small cell lung cancer (NSCLC).

## Methods

### Study population

Two hundred and twenty-one patients who were pathologically diagnosed with NSCLC and received surgical treatment at the Roswell Park Cancer Institute (RPCI) from 1995 to 1999 were included in the current study. These patients were identified and selected based on the following criteria: 1) no previous history of cancer; 2) no other primary cancers diagnosed within one month of the current diagnosis; 3) initial surgery for NSCLC at RPCI; 4) pathology report of complete removal of gross tumor at surgery; and 5) minimum survival of one month after surgery. After examination of patient follow-up data and tumor immunostaining, 53 patients were excluded due to inadequate tumor specimen or poor staining, and 40 were excluded due to causes of death not related to lung cancer, leaving a total of 128 patients in the current analysis. The pathological stage of these patients included 75 stage I, 31 stage II, and 22 stage IIIA, and histological classification included 77 adenocarcinomas, 39 squamous cell carcinomas, 5 large cell tumors, and 7 tumors with mixed histological differentiation. The majority of tumors were poorly differentiated (60.2%), and the remainder was well and moderately differentiated (39.8%). This study was approved by the Institutional Review Board at RPCI.

### Tumor and patient data

Patient clinicopathological data were retrieved from medical records. These data included sex, age, race, lifetime smoking history, family history of lung cancer, date of diagnosis, date of surgery, adjuvant chemotherapy, pathological TNM stage of tumor, histology, grade, performance status, weight loss, and date of last follow-up. Patient performance status was scored according to the Zubrod Performance Scale [34]. Weight loss was defined as more than 5% of weight loss within three months prior to the diagnosis. The date of last follow-up was the date of death for deceased patients, and the last date of contact for living patients by the Department of Medical Record at RPCI.

### Detection and scoring methods

Tissue specimens obtained from diagnostic or surgical procedures were fixed in neutral buffered formalin (10% vol/vol formalin in water; pH = 7.4), and embedded in paraffin wax. Consecutive 5-μm thick tumor sections were cut and mounted on charged glass slides (Superfrost Plus; Fisher Scientific, Rochester, NY). Sections were warmed at 60–70°C for 20 minutes, and then were deparaffinized in xylene, and rehydrated in 100% and 95% ethanol. Antigen retrieval was performed for Ki-67 and gelsolin staining. Sections to be treated with antibodies against Ki-67 or gelsolin were microwaved twice for 10 minutes in citrate buffer, followed by cooling to room temperature for 20 minutes. Our previous study showed good immunohistochemical staining for anti-MCM2 antibody without antigen retrieval [9]. An affinity-purified rabbit antibody against the N-terminal region of MCM2 was used for MCM2 staining [9]. Since the epitope recognized by anti-Ki-67 antibody is usually destroyed during formalin fixation and paraffin embedding [35], we utilized MIB-1 mouse monoclonal antibody to detect Ki-67 antigen (Immunotech Inc., Westbrook, ME). MIB-1 antibody was developed using a recombinant partial structure of the Ki-67 antigen as immunogen, and it is widely used as a Ki-67 equivalent monoclonal antibody [35]. It can detect Ki-67 antigen in formalin-fixed, paraffin-embedded tissues through antigen retrieval, and the immunostaining pattern in fresh frozen tissue is identical to that of anti-Ki-67 antibody [36]. The MCM2 antibody, generated as described by Todorov et al. [9], and MIB-1 antibody were incubated for 60 and 30 minutes at room temperature and used at dilutions of 1:500 and 1:100, respectively, for optimal staining effect. The avidin-biotin detection method was used on a Ventana Automated System (Ventana Medical Systems, Tucson, AZ). An irrelevant rabbit antiserum was used as a negative control. The percentage of nuclei stained for both antibodies in cancer cells was evaluated by two investigators (D.T. and J.S.J.B.).

For gelsolin immunoassay, the primary anti-gelsolin monoclonal antibody GS-2C4 (Sigma Chemical Co., St. Louis, MO) was titrated at the optimal concentration of 1:1000 in phosphate buffered saline. The antibody was manually added to each section and incubated at 42°C for 32 minutes. All other staining procedures were performed by the Ventana 320 Automated Slide Staining System (Ventana Medical Systems, Tucson, AZ), using the ABC (avidin-biotin-conjugate) immunoperoxidase protocol with goat anti-mouse (peroxidase-conjugated) as secondary antibody. In order to assess the performance of the overall staining process, normal lung tissue sections from clinical biopsies were used as external positive and negative staining controls in each run. Negative controls were exposed to all steps in the Ventana System except the primary antibody. Stained slides were evaluated and scored simultaneously by two research investigators (D. F. T. and J. Y.) under a multi-head light microscope. The percentage of tumor cells was determined at four staining intensities (0, 1+, 2+, or 3+). Pulmonary macrophages (3+), fibroblasts (2+), and normal lung epithelial cells (1+ to 2+) adjacent to tumor cells (if available) were used as scoring references [31,37]. The percentage of tumor cells at four staining intensities was counted. To assess the inter-observer variability of scoring, approximately half of the cases (n = 56) were randomly selected and reviewed by two pathologists (D. F. T. and J. G.) independently, which yielded an inter-observer agreement of 81%.

Based on the current literature and our experiences, the phenotypic expression features of MCM2, Ki-67, and gelsolin warranted different scoring systems for these three markers. MCM2 and Ki-67 expression was defined as low if less than 25% of tumor cells showed staining in nuclei in a tumor section. This definition was used according to the commonly used cutoff values ranging from 20–40% in non-small cell lung cancer and other human cancers in the current literature [38-41] and also based on the examination of our staining data. For gelsolin expression, an index was calculated to account for non-uniform expression in terms of staining intensity and number of tumor cells at each intensity. The index was calculated by multiplying the percentage of tumor cells by the corresponding staining intensity, as described previously [33]. Since the index distribution was skewed, the median (score = 40) rather than the mean (score = 63) value was used as the cut-point for the dichotomization of gelsolin expression (low if index ≤40, and high if index >40).

### Statistical methods

The Student's t-test and chi-square test were used to examine the association between the expression of MCM2, Ki-67 and gelsolin and patient clinicopathological characteristics. For smoking status, stage, and grade, which were measured as ordinal variables, we utilized Kendall's tau-b test for the chi-square analysis to take into account the ordinal measurement. Exact tests were used when cell frequencies were less than 10. The examination of proportional hazards showed that the hazards were proportional for MCM2 and gelsolin over time, but non-proportional for Ki-67, thus we used Cox proportional hazards regression to compute the relative risk (RR) of death for MCM2 and gelsolin, and used the time-dependent Cox regression to evaluate the prognostic value of Ki-67 [42]. Kaplan-Meier survival analysis was used to generate survival curves. The log rank test was utilized to examine the difference of the accumulated survival function for MCM2 and gelsolin, and Breslow test was used to test the difference of survival function for Ki-67, which takes into account the number of cases in risk at each time point over the follow-up period. Age and factors that demonstrated a significant prognostic effect in the univariate model (stage, adjuvant chemotherapy, family history of lung cancer, and smoking history) were adjusted for RR estimation in the multivariate Cox regression model. All analytic procedures were performed using SPSS statistical software version 13.0 (SPSS, Inc., Chicago, IL).

## Results

Patient characteristics are summarized in Table 1. The majority of patients were Caucasian (93.8%). There were more male (54.7%) than female patients (45.3%). The majority of patients were former smokers (50%) or current smokers (41%). About one third of patients had a positive family history of lung cancer. The mean and median survival time among deceased patients was 29.4 and 26.5 months, respectively (range: 1.5–84.7 months). The mean and median follow-up time for living patients was 68.3 and 67.0 months, respectively (range: 42.7–128.4 months). Forty-nine patients (38.3%) were alive at the last contact on December 30^th^, 2003.

**Figure 1:**
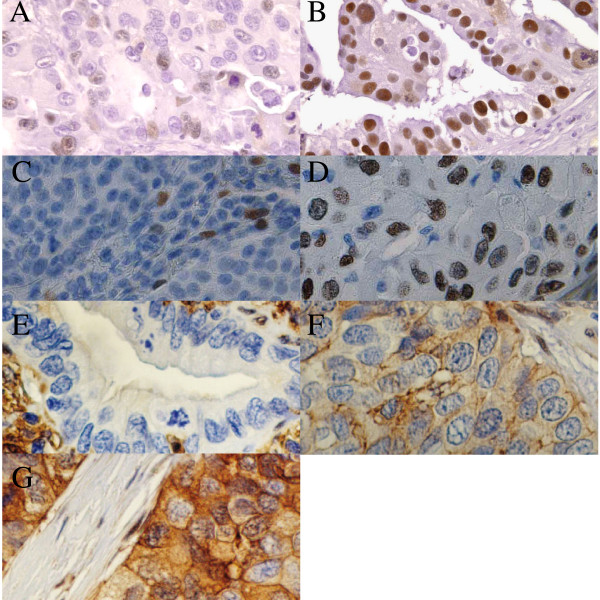
Representative images showing immunostaining of proliferation and motility markers (MCM2, Ki-67 and gelsolin) in NSCLC. A: low MCM2; B: high MCM2; C: negative Ki-67 (note: a few active lymphocytes are positive); D: high Ki-67; E: negative gelsolin; F: low gelsolin; G: high gelsolin. Magnification ×400 in D, E, F, and G; ×200 in A, B, and C.

The phenotypic expressions of MCM2, Ki-67, and gelsolin are shown in Figure 1, which depicts examples of negative expression (C and E), low expression (A and F), and high expression (B, D and G), respectively. Examination of MCM2, Ki-67, and gelsolin expression showed that 61.7%, 78.1% and 32.8% of tumors exhibited high expression of these three proteins, respectively (Table 1). Patient characteristics stratified by levels of MCM2, Ki-67 and gelsolin expression are shown in Table 2. None of the clinicopathological factors showed a significant association with Ki-67 expression. For MCM2, high expression was associated with sex (male patients: 64.6% vs. 38.8%), histology (squamous carcinomas: 45.6% vs. 6.1%), and grade (poorly differentiated tumors: 68.4% vs. 46.9%). For gelsolin, high expression was associated with sex (female patients: 66.7% vs. 51.2%), and histology (squamous carcinomas: 38.1% vs. 26.7%).

Univariate analysis of the prognostic significance of MCM2, Ki-67, and gelsolin showed higher levels of gelsolin were significantly associated with an elevated risk of death compared to low levels of gelsolin (RR = 1.89, 95% CI = 1.20–2.98, p = 0.006), and higher levels of MCM2 were associated with a non-significant increased risk of death compared to low levels of MCM2 (RR = 1.51, 95% CI = 0.94–2.41, p = 0.09). Ki-67 expression did not display apparent prognostic effect in this study sample (RR = 0.70, 95% CI = 0.27–1.86, p = 0.47). After controlling for age, stage, adjuvant chemotherapy, family history of lung cancer, and smoking history, the significant association remained between high levels of gelsolin and elevated risk of death (RR = 1.89, 95% CI = 1.17–3.05, p = 0.01). The prognostic effect of MCM2 was attenuated after controlling for confounders (RR = 1.36, 95% CI = 0.84–2.20, p = 0.22) (Table 3).

**Figure 2:**
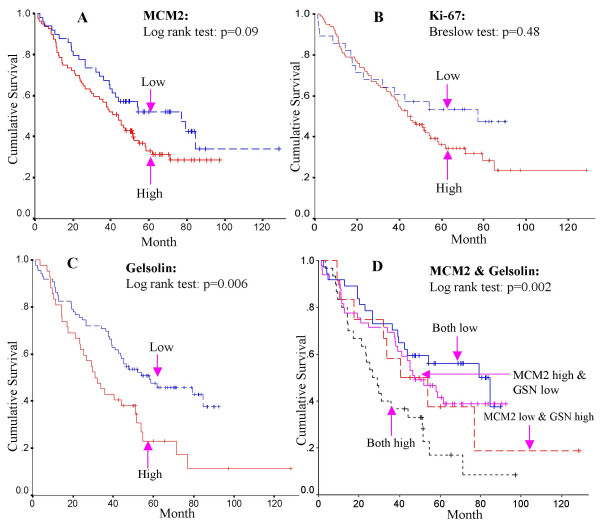
Kaplan-Meier Survival Curves for MCM2, Ki-67, and Gelsolin Expressions among 128 Patients with NSCLC. A: MCM2; B: Ki-67; C: Gelsolin; D: MCM2 & Gelsolin. Tick marks represent censored cases.

Composite analysis of MCM2 and gelsolin expression revealed that higher expression in MCM2 and gelsolin was significantly associated with higher risk of death compared to low expression in both biomarkers (adjusted RR = 2.32, 95% CI = 1.21–4.45, p = 0.01) (Table 3, Figure 2). Since the hazards function was not proportional for Ki-67, we did not analyze the combined prognostic effect of Ki-67 with MCM2 and gelsolin in the Cox Proportional Hazards Regression. Survival curves generated from Kaplan-Meier survival analysis are illustrated in Figure 2. The curves confirmed the results from Cox regression that patients with higher levels of MCM2 and gelsolin experienced shorter survival time than patients with low levels of MCM2 and gelsolin.

## Discussion

The relative balance between cell growth and cell death is altered in cancers, with the balance tipped towards uncontrolled proliferative capacity in cancer cells [1,43]. In addition, some cancer cells acquire a markedly enhanced capacity to move, thereby predisposing them to invasion and dissemination [44,45]. Findings from the present study showed that tumors exhibiting higher levels of expression in MCM2, a marker of proliferation, and gelsolin, a marker of motility, conferred a higher risk of death in NSCLC.

MCM proteins are essential replication initiation factors that ensure the accurate replication of DNA in the cell cycle. Through accurate binding to chromatin during G_1 _phase and detaching from chromatin during S phase, MCM proteins restrict DNA synthesis to only once per cell cycle [3,4,6]. Only licensed origins containing MCM proteins can initiate a pair of replication forks, and once initiation occurs at an origin, the bound MCM proteins are displaced so that the origin cannot fire again [46]. MCM proteins may also function as the helicase that unwinds DNA ahead of each replication fork [47]. Evidence from different organisms and cells indicates that both permanently arrested cells and those in G_0 _phase have lost expression of MCM proteins and are functionally unlicensed [48-50]. The replication initiation function of MCM proteins in cell cycle implies that they may be valuable markers for proliferation and prognosis in human cancers. Todorov et al. studied MCM2 expression in normal and tumor human tissues and found MCM2 was expressed significantly different in specimens examined, with expression of 97% in tumors but only 27% in normal tissues. Furthermore, the expression level in normal tissues was lower than in tumor tissues, indicating that MCM2 reflects cell proliferation [9]. Dudderidge et al. studied MCM2, geminin, and Ki-67 in renal cell carcinoma and found that MCM2 expression increased dramatically with increasing grade [41]. Hashimoto et al. investigated MCM2 and Ki-67 expression in lung adenocarcinomas and found that the co-expression of MCM2 and Ki-67 at higher levels were significantly associated with poorer survival [39]. The prognostic value of MCM2 was also reported in bladder cancer and oligodendroglioma [40,51].

Ki-67 is expressed in all stages of the cell cycle except G_0 _phase, making it a valuable measurement for cell proliferation [12]. In a number of cancers, elevated Ki-67 expression has been found associated with higher aggressiveness and invasiveness [52-54]. The prognostic value of Ki-67 has been observed in cancers of breast, prostate, cervix, and soft tissue [21]. In a comprehensive review, Pugsley et al. noted that higher Ki-67 index was significantly associated with poorer survival in NSCLC in the majority of univariate analyses and in approximately half of the multivariate analyses [38]. In our study, we did not observe the prognostic effect of Ki-67 expression in both the univariate and multivariate analysis, which may be partially due to the limited sample size. The small sample size also prevented additional examination of the prognostic value of MCM2, Ki-67 and gelsolin in subgroup patients. As Pugsley et al. pointed out, factors such as patient heterogeneity, retrospective nature of study design, quantification of immunohistochemistry, and cutoff value may contribute to the inconsistent findings for the prognostic value of Ki-67 NSCLC in the literature [38].

In addition to proliferation, elevated motility is essential for tumor cells to invade and disseminate [44,45,55,56]. Highly metastatic cells typically exhibit enhanced locomotion capacity compared to less metastatic cells [44,45,57,58]. Various studies have consistently shown that higher levels of motility are associated with higher rates of tumor metastasis or shorter survival time [31-33,59-62]. The motility facilitating function of gelsolin has been widely observed in animal models [26-29]. We and others have showed that higher levels of gelsolin are associated with poorer survival in breast and lung cancer [31-33]. Some studies failed to find a prognostic significance for gelsolin. However, these studies are generally subject to small sample size or inadequate follow-up time [37,63-65]. As a generalization, the gelsolin content of the cells in most cancers is found to be down-regulated [37,64-66], but in a number of studies, researchers found that high gelsolin expression is inversely associated with survival time [31-33]. For example, we found that tumors with high and heterogeneous expression of gelsolin conferred the highest risk of death from NSCLC in patients with this disease [33]. Shieh et al. found that the most potent predictor of poor prognosis was high focal gelsolin expression [31]. Increased expression of gelsolin may facilitate a subset of tumor cells with increased motility, thereby enhancing its capability and probability of invading adjacent tissues and metastasis to remote organ sites [32,33].

## Conclusion

In summary, higher expression in MCM2 and gelsolin was significantly associated with poorer prognosis in patients with NSCLC, which suggests that higher tumor proliferation and motility may be important in the prognosis of NSCLC. Ki-67 did not display apparent prognostic value in NSCLC in our study sample. Composite application of proliferation and motility markers may be more valuable than use of a single marker in assessing tumor prognosis. This combinatorial approach may also prove valuable in assessing response to anticancer treatment and the subsequent clinical outcomes.

## Abbreviations

ABC: avidin-biotin-conjugate; GSN: gelsolin; MCM: minichromosome maintenance; NSCLC: non-small cell lung cancer; RPCI: Roswell Park Cancer Institute; RR: relative risk

## Competing interests

The author(s) declare that they have no competing interests.

## Authors' contributions

JY carried out and coordinated the study, conducted IHC and data analysis, and drafted up the manuscript. NR participated in the study design, interpretation of data, and revision of manuscript. KBM participated in the study design, interpretation of data, and revision of manuscript. HLA developed gelsolin scoring system, interpreted data, and revised manuscript. SJA collected the tumor and clinicopathological data. JH interpreted data and revised manuscript. JG and JSJB performed IHC examinations of tumor specimens and revised manuscript. DT participated in the study design, IHC examinations of tumor specimens, interpretation of data, and revision of manuscript. All authors read and approved the final manuscript.

## Pre-publication history

The pre-publication history for this paper can be accessed here:



## Figures and Tables

**Table 1:** Characteristics of 128 Non-small Cell Lung Cancer Patients, RPCI, 1995–1999

**Characteristics**	**N = 128 (%)**
**Sex**	
Male	70 (54.7%)
Female	58 (45.3%)
**Race**	
Caucasians	120 (93.8%)
Non-Caucasians	8 (6.2%)
**Age**	
Mean (SD)	64.2 (10.7)
**Smoking status**	
Never	11 (8.6%)
Former	64 (50.0%)
Current	53 (41.4%)
**Pack-years of smoking**^(a)^	
Mean (SD)	51.4 (27.0)
**Family history of lung cancer**	
No	87 (68.0%)
Yes	41 (32.0%)
**Zubrod Performance status**	
Without symptom	87 (68.0%)
With symptom	41 (32.0%)
**Weight loss**^(b)^	
No	110 (85.9%)
Yes	18 (14.1%)
**Vital status**	
Alive	49 (38.3%)
Dead	79 (61.7%)
**Pathologic TNM stage**	
I	75 (58.6%)
II	31 (24.2%)
IIIA	22 (17.2%)
**Histology**	
Adenocarcinoma	77 (60.2%)
Squamous	39 (30.5%)
Large cell	5 (3.9%)
Others	7 (5.5%)
**Grade**	
Well differentiation	3 (2.3%)
Moderate differentiation	48 (37.5%)
Poor differentiation	77 (60.2%)
**Receiving adjuvant chemotherapy**	
No	109 (85.2%)
Yes	19 (14.8%)
**MCM2**	
Low	49 (38.3%)
High	79 (61.7%)
**Ki-67**	
Low	28 (21.9%)
High	100 (78.1%)
**Gelsolin level**	
Low	86 (67.2%)
High	42 (32.8%)

(a). Pack-years of smoking: packs of cigarette smoked per day multiplied by years of smoking;

(b). Weight loss: ≥ 5% of total weight loss within 3 months prior to diagnosis.

**Table 2:** Association between MCM2, Ki-67, and Gelsolin and Clinicopathological Characteristics in 128 Patients with NSCLC, RPCI, 1995–1999

Clinicopathological Characteristics	Low MCM2 (n = 49)	High MCM2 (n = 79)	Low Ki-67 (n = 28)	High Ki-67 (n = 100)	Low GSN (n = 86)	High GSN (n = 42)
**Gender**						
Male	19 (38.8%)	51 (64.6%)**	13 (46.4%)	57 (57.0%)	42 (48.8%)	14 (33.3%)*
Female	30 (61.2%)	28 (35.4%)	15 (53.6%)	43 (43.0%)	44 (51.2%)	28 (66.7%)
**Race**						
Caucasians	47 (95.9%)	73 (92.4%)	26 (92.9%)	94 (94.0%)	83 (96.5%)	37 (88.1%)
Non-Caucasians	2 (4.1%)	6 (7.6%)	2 (7.1%)	6 (6.0%)	3 (3.5%)	5 (11.9%)
**Mean age (SD) Smoking status**	64.7 (9.2)	63.9 (11.6)	63.0 (10.9)	64.6 (10.7)	64.8 (9.6)	63.2 (12.8)
Current	18 (36.7%)	35 (44.3%)	11 (39.3%)	42 (42.0%)	34 (39.5%)	19 (45.2%)
Former	27 (55.1%)	37 (46.8%)	14 (50.0%)	50 (50.0%)	43 (50.0%)	21 (50.0%)
Never	4 (8.2%)	7 (8.9%)	3 (10.7%)	8 (8.0%)	9 (10.5%)	2 (4.8%)
**Mean pack-years (SD)**^(a)^	47.9 (30.2)	46.4 (29.4)	45.6 (36.4)	47.4 (27.6)	44.6 (28.8)	51.9 (31.0)
**Family history**						
No	35 (71.4%)	52 (65.8%)	23 (82.1%)	64 (64.0%)	58 (67.4%)	29 (69.0%)
Yes	14 (28.6%)	27 (34.2%)	5 (17.9%)	36 (36.0%)	28 (32.6%)	13 (31.0%)
**Pathology Stage**						
I	30 (61.2%)	45 (57.0%)	16 (57.1%)	59 (59.0%)	52 (60.5%)	23 (54.8%)
II	15 (30.6%)	16 (20.2%)	8 (28.6%)	23 (23.0%)	22 (25.6%)	9 (21.4%)
IIIA	4 (8.2%)	18 (22.8%)	4 (14.3%)	18 (18.0%)	12 (14.0%)	10 (23.8%)
**Tumor size**						
≤3 cm	22 (45.8%)	27 (34.2%)	12 (42.9%)	37 (37.4%)	35 (41.2%)	14 (33.3%)
>3 cm	26 (54.2%)	52 (65.8%)	16 (57.1%)	62 (62.6%)	50 (58.8%)	28 (66.7%)
**Lymph node status**						
Negative	34 (70.8%)	47 (59.5%)	18 (64.3%)	63 (63.6%)	58 (68.2%)	23 (54.8%)
Positive	14 (29.2%)	32 (40.5%)	10 (35.7%)	36 (36.4%)	27 (31.8%)	19 (45.2%)
**Histology**						
Adenocarcinoma	41 (83.7%)	36 (45.6%)**	20 (71.4%)	57 (57.0%)	56 (65.1%)	21 (50.0%)*
Squamous	3 (6.1%)	36 (45.6%)	6 (21.4%)	33 (33.0%)	23 (26.7%)	16 (38.1%)
Large cell	2 (4.1%)	3 (3.8%)	1 (3.6%)	4 (4.0%)	5 (5.8%)	0 (0.0%)
Others	3 (6.1%)	4 (5.1%)	1 (3.6%)	6 (6.0%)	2 (2.3%)	5 (11.9%)
**Grade**						
Well differentiation	1 (2.0%)	2 (2.5%)*	2 (7.1%)	1(1.0%)	1(1.1%)	2(4.8%)
Mod. differentiation	25 (51.1%)	23 (29.1%)	13 (46.4%)	35 (35.0%)	33 (38.4%)	15 (35.7%)
Poor differentiation	23 (46.9%)	54 (68.4%)	13 (46.4%)	64 (64.0%)	52 (60.5%)	25 (59.5%)
**Performance Status**						
Without symptom	32 (65.3%)	55 (69.6%)	21 (75.0%)	66 (66.0%)	61 (70.9%)	26 (61.9%)
With symptom	17 (34.7%)	24 (30.4%)	7 (25.0%)	34 (34.0%)	25 (29.1%)	16 (38.1%)
**Weight loss**^(b)^						
No	43 (87.8%)	67 (84.8%)	24 (85.7%)	86 (86.0%)	76 (88.4%)	34 (81.0%)
Yes	6 (12.2%)	12 (15.2%)	4 (14.3%)	14 (14.0%)	10 (11.6%)	8 (19.0%)
**Mean survival in month (SD)**	49.8 (28.0)	40.8 (25.7)	50.0 (31.3)	42.7 (25.4)	48.0 (26.6)	36.7 (26.1)*

(a). Pack-years of smoking: packs of cigarette smoked per day multiplied by years of smoking;

(b). Weight loss: ≥ 5% of total weight loss within 3 months prior to diagnosis.

* p ≤ 0.05; **: p ≤ 0.01

**Table 3:** Cox Regression of Risk of Death in Association with MCM2, Ki-67, and Gelsolin in 128 Patients with NSCLC, RPCI, 1995–1999

**Variables**	**Crude RR (95%CI)**	**P value**	Adjusted RR (95%CI)*	**P value**
**MCM2**^†^				
Low (n = 49)	1.00		1.00	
High (n = 79)	1.51 (0.94–2.41)	0.09	1.36 (0.84–2.20)	0.22
				
**Ki-67**^‡^				
Low (n = 28)	1.00		1.00	
High (n = 100)	0.70 (0.27–1.86)	0.47	0.59 (0.22–1.61)	0.30
				
**Gelsolin**^†^				
Low (n = 86)	1.00		1.00	
High (n = 42)	1.89 (1.20–2.98)	0.006	1.89 (1.17–3.05)	0.01
				
**Gelsolin and MCM2**^†^				
Both low (n = 37)	1.00		1.00	
GSN low, MCM2 high (n = 49)	1.34 (0.74–2.42)	0.33	1.34 (0.73–2.45)	0.34
GSN high, MCM2 low (n = 12)	1.62 (0.70–3.73)	0.26	2.08 (0.87–4.98)	0.10
GSN high, MCM2 high (n = 30)	2.59 (1.39–4.80)	0.003	2.32 (1.21–4.45)	0.01

* Adjusted for age, stage, adjuvant chemotherapy, family history of lung cancer, and smoking history.

† Results from Cox Proportional Hazards Regression.

‡ Results from time-dependent Cox Regression.
